# The Association of Breastfeeding With Childhood Asthma: A Case-Control Study From India

**DOI:** 10.7759/cureus.19810

**Published:** 2021-11-22

**Authors:** Peri Harish Kumar, Amit Devgan

**Affiliations:** 1 Department of Paediatrics, Armed Forces Medical College, Pune, IND

**Keywords:** exclusive breastfeeding, breastfeeding duration, childhood asthma, peak expiratory flow rate, breastfeeding

## Abstract

Background

The role of breastfeeding in childhood asthma has long been controversial. The majority of research pertains to developed countries with scant literature available in a developing country like India, where a different asthma phenotype is prevalent. This study examined the association of breastfeeding duration and exclusiveness with childhood asthma and its severity, as measured by peak expiratory flow rate (PEFR) in India.

Methodology

We conducted a matched case-control study in Pune, India. A total of 180 children with asthma (cases) and 180 without the disease (controls) were included. A standardized questionnaire recorded demographics and medical and breastfeeding history. PEFR readings were obtained from each child. Conditional logistic regression and linear regression were used to explore the association of breastfeeding with asthma and PEFR, respectively.

Results

The median duration of breastfeeding among cases was [5 (2.5-10)] months as compared to controls [9 (3.5-16.8)] months. The prevalence of exclusive breastfeeding among mothers was 60% (50% among cases and 69% among controls). Exclusive breastfeeding was associated with a 46% lower likelihood of having asthma with a probability (p-value) of 0.025 where the odds ratio (OR) was 1.85, with a 95% confidence interval (CI) of 1.08 to 3.16. Breastfeeding duration was significantly associated with a lower likelihood of having asthma (p = 0.001) (OR 0.87; 95% CI 0.79-0.94). One-month increase in the duration of breastfeeding was associated with a 23% reduced risk of the disease. The odds of maternal asthma [21.4 (4.22-109.36)], paternal smoking [1.44 (0.22-0.86)], and maternal smoking [5.14 (1.78-14.80)] were higher among children with asthma as compared to children without asthma. The weight of the child and duration of breastfeeding were negatively associated with PEFR. Maternal asthmatic history, associated allergies, paternal smoking, and parents’ education were positively associated with PEFR for the overall sample.

Conclusion

Prolonged and exclusive breastfeeding was found to be a protective factor against the development of asthma. Promotion of breastfeeding and smoking cessation should be a priority in the control of childhood asthma. Further research should be conducted to explore the negative correlation between duration and frequency of breastfeeding and PEFR.

## Introduction

With an estimated 339 million (4.3%) sufferers worldwide, childhood asthma poses a substantial public health challenge. It is also among the leading causes of chronic morbidity among children, evidenced by the vast disability-adjusted life years. In India, the estimated prevalence of asthma ranges from 2% to 23%, with wide confidence intervals owing to varied study methodologies and different population and environmental characteristics [1]. Asthma is a multifactorial disease. Known risk factors include male gender, low birth weight, pre-term birth, poor parental socio-economic status, ethnicity, family history of asthma or atopy, and parental smoking [2-4].

Extensive research was conducted on the role of breastfeeding as a risk factor in childhood asthma. Human breast milk is highly beneficial for the development of the immune system due to milk components, such as antigens, immunoglobulin A (IgA), polyamines, polyunsaturated fatty acids, chemokines, and cytokines [5,6]. Some studies have found that it contains live microbes, which contribute to the gut microbiota, leading to a reduced incidence of asthma among children [7,8]. Additionally, suckling promotes pulmonary function as it is a form of exercise that builds strong pulmonary muscles [9]. However, several studies have reported either no protective association between breast milk and asthma or have observed increased asthma risk among children with breastfeeding [10-13]. Therefore, the role of breastfeeding in reducing the risk of childhood asthma has remained controversial.

Most of the studies were conducted in developed countries where the prevalent asthma phenotype is different from those prevalent in a developing country like India. Studies about the association in our country are very scanty. Therefore, we undertook this study considering the public health importance of this issue and the severe paucity of literature on the subject in our country. We examined the effect of breastfeeding on the risk of developing childhood asthma and its severity (as measured by the peak expiratory flow rate [PEFR] levels) among children aged 6-12 years. The evaluating parameters among breastfeeding were the duration and type of breastfeeding (exclusive vs. non-exclusive).

## Materials and methods

Design and setting

This study was conducted in the pediatric outpatient department of Command Hospital, Pune, India, in Wanowrie and National Institute of Bank Management (NIBM) areas (vicinity areas within 5 km of the hospital) from August 2012 to January 2014. A matched case-control design was adopted for the study.

Participants

The participants comprised children in the age group of 6-12 years. Cases were children with asthma, recruited from the pediatric outpatient department of Command Hospital, Pune in India. Eligibility criteria for cases included the diagnosis of asthma by a certified pediatrician, no other comorbidities, and regular follow-up for one year. Controls were healthy children recruited from government schools in Wanowrie and NIBM areas. They were matched for age, weight, and height with the cases. The case to control ratio for the study was 1:1.

Data collection and evaluation parameters

A standardized questionnaire was used to collect information about the child's age, sex, weight, height, gestational age at birth, presence or absence and duration of asthma, familial history of asthma, socio-economic status, any other comorbidity, breastfeeding duration, and the exclusivity, parental education, and smoking. The format of the standard questionnaire is presented in the Appendix.

The evaluating parameters for the exposure (breastfeeding) included its duration and exclusivity. The following operational definitions were considered for the assessment of exposure:

(i) Exclusive breastfeeding: Mode of feeding that involves "human milk only (including donor human milk) for at least the first six months of life without any other food, water, or other fluids, although vitamin and mineral supplements or medicine syrups are allowed" [14]. Feeding inclusive of other sources and breastfeeding for less than six months was considered non-exclusive breastfeeding.

(ii) Duration: For analytical purposes, the duration of breastfeeding, regardless of exclusivity, was categorized into three groups: never breastfed, breastfed for less than six months, and breastfed for six months or more.

The evaluating parameters for the outcome (childhood asthma) included its prevalence and severity. The severity of asthma was assessed by measuring the PEFR of the children.

Participant selection

**Figure 1:**
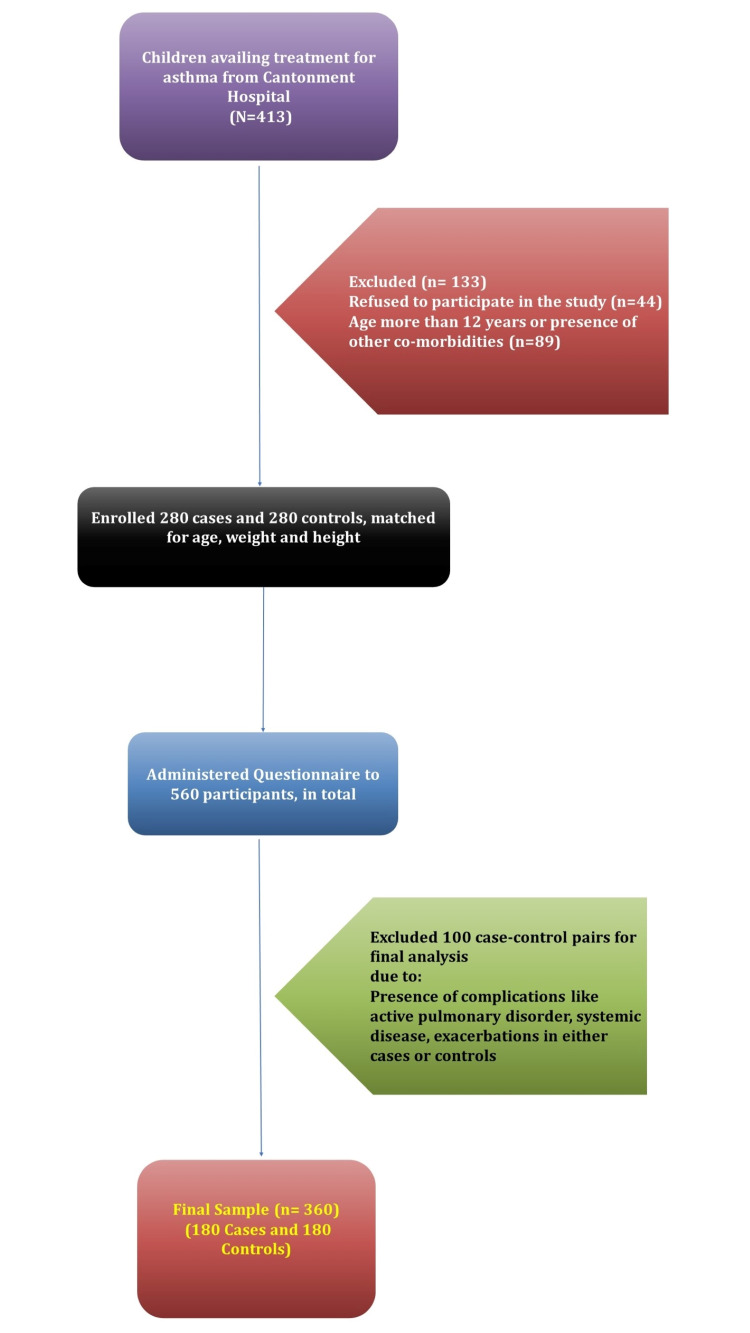
A flow chart showing the selection of participants for the study.

From the previous two years' hospital records, we found that 413 children aged 6-12 years were seeking treatment from our hospital for more than one year for asthma. We contacted them during their visit to the pediatric outpatient department. A total of 133 children were then excluded from the study due to various reasons such as refusal to participate (n = 44), age greater than 12 years, or presence of other comorbidities (n = 89). A total of 280 controls were then identified, matching age, height, and weight to the cases population. A written, informed consent was obtained from the parents or guardians of the participating population. An in-person questionnaire was then distributed, and exhaustive detailing was done to explain how to fill them. On reviewing the questionnaires, it was observed that complications like active pulmonary disorder, another systemic disease, or acute asthma were present in some cases or controls. As a result, 100 further case-control pairs were excluded from the final analysis. A final sample of 180 cases with 180 controls was arrived at. A flow chart showing the selection of participants is provided in Figure 1.

PEFR was then measured for each child in the study. Three consecutive PEFR readings were taken using a Mini-Wright peak flow meter, and the highest PEFR value for each child was recorded.

This study was approved by the Institutional Ethics Clearance Committee of the Armed Forces Medical College, Pune, India (2012-02167) on January 23, 2012.

## Results

Out of 360 children, 180 were asthmatics enrolled as cases, and the rest (180) were normal controls, matched for age, height, and weight. Normality tests indicated the non-normal distribution of continuous variables; hence, the median was used as a summary measure instead of the mean, as shown in Table 1.

**Table 1:** Shapiro-Wilk test findings to test distribution of continuous data. PEFR, peak expiratory flow rate.

Variable	Group	Shapiro-Wilk* *test	
		Statistic	p-value
Height (cm)	Control	0.982	0.020
	Case	0.990	0.217
Weight (kg)	Control	0.971	0.001
	Case	0.959	<0.01
Age (years)	Control	0.943	<0.01
	Case	0.944	<0.01
PEFR (L/min)	Control	0.993	0.488
	Case	0.929	<0.01
Duration of breastfeeding (months)	Control	0.891	<0.01
	Case	0.867	<0.01

Socio-demographic profile and PEFR

There were 87 (48%) and 96 (53%) females among cases and controls, respectively. The median age [9 (8-10) years], height [133 (127-139) cm], and weight [28 (26-31) kg] of children were the same in both groups. However, the median PEFR was significantly less among cases [126 (113-141.8) l/min] than in controls [197.5 (176-214) l/min], with a p-value of <0.01.

Medical and family history: bivariate analysis

The distribution of illiterate parents was significantly more among cases (n = 100, 56%) than controls (n = 77, 43%). Similarly, significantly more children among cases (n = 85, 47%) had history of maternal asthma as compared to controls (n = 65, 36%). The details are shown in Table 2.

**Table 2:** Medical and family history of study participants.

Variable	Attributes	Case (n = 180)		Control (n = 180)		Total (N = 360)		p-value
		n	%	n	%	n	%	
Parent education	Illiterate	100	56	77	43	177	49.2	0.02
	Literate	80	44	103	57	183	50.8	
Timing of birth	Pre-term	36	20	39	22	75	20	0.80
	Term	144	80	141	78	285	79.2	
Any associated allergy	Yes	60	33	63	35	123	34.2	0.82
	No	120	67	117	65	237	65.8	
Paternal asthma	Yes	59	33	64	36	123	34.2	0.66
	No	121	67	116	64	237	65.8	
Sibling asthma	Yes	61	34	73	41	134	37.2	0.23
	No	119	66	107	59	226	62.8	
Maternal asthma	Yes	85	47	65	36	150	41.7	0.04
	No	95	53	115	64	210	58.3	
Paternal smoking	Yes	111	62	123	68	234	65	0.22
	No	69	38	57	32	126	35	
Maternal smoking	Yes	27	15	15	8	42	11.7	0.07
	No	153	85	165	92	318	88.3	

Details of exposure of interest (breastfeeding): bivariate analysis

It was found that the median duration of breastfeeding among cases [5 (2.5-10) months] was significantly less than that of controls [9 (3.5-16.8) months] with a p-value of 0.04. The proportion of children who were exclusively breastfed was significantly higher among controls (n = 125, 69%) as compared to cases (n = 90, 50%), as shown in Table 3. However, the distribution of children based on breastfeeding frequency was not found to be significantly different between the two groups (p = 0.07).

**Table 3:** Details of breastfeeding among cases and controls.

Variable	Attributes	Case (n = 180)		Control (n = 180)		Total (N = 360)		p-value
		n	%	n	%	n	%	
Exclusive breastfeeding	Yes	90	50	125	69	208	59.7	<0.01
	No	90	50	55	31	138	40.3	
Breastfeeding frequency	Never	8	4.4	4	2.2	12	3.3	0.07
	Less than six months	101	56.1	85	47.2	186	51.7	
	Six months or more	71	39.4	91	50.6	162	45	

Factors affecting asthma among children: conditional logistic regression

Duration of breastfeeding, the exclusivity of breastfeeding, parental education, and history of any associated allergy, maternal asthma, and paternal and maternal smoking were significantly associated with asthma among cases and controls, after controlling for confounding. See Table 4 for details.

**Table 4:** Regression model to assess the factors associated with asthma. OR, odds ratio; CI, confidence interval.

Variable	Attributes	Unadjusted OR (95% CI)	p-value	Adjusted OR (95% CI)	p-value
Duration of breastfeeding (months)	5 (2.5-10) months vs. 9 (3.5-16.8) months	0.94 (0.91-0.97)	<0.01	0.87 (0.79-0.94)	0.001
Exclusive breastfeeding	Yes	1		1	
	No	2.27 (1.48-3.50)	<0.01	1.85 (1.08-3.16)	0.025
Breastfeeding frequency	Six months or more	1		1	
	Less than six months	1.188 (0.890-1.586)	0.241	0.45 (0.16-1.25)	0.13
	Never	2.00 (0.602-6.642)	0.258	0.62 (0.09-3.87)	0.61
Parent education	Illiterate	1		1	
	Literate	0.60 (0.39-0.91)	0.02	0.09 (0.03-0.27)	<0.01
Timing of birth	Pre-term	1		1	
	Term	1.11 (0.67-1.84)	0.7	0.96(0.32-2.87)	0.95
Any associated allergy	Yes	1.08 (0.70-1.67)	0.74	0.040 (0.007-0.203)	<0.01
	No	1		1	
Paternal asthma	Yes	1.13 (0.73-1.75)	0.58	0.17 (0.02-1.31)	0.09
	No	1		1	
Sibling asthma	Yes	1.33 (0.87-2.04)	0.19	1.26 (0.72-1.34)	0.99
	No	1		1	
Maternal asthma	Yes	0.63 (0.41-0.96)	0.03	21.4 (4.22-109.36)	<0.01
	No	1		1	
Paternal smoking	Yes	1.75 (0.48-1.15)	0.19	1.44 (0.22-0.86)	0.02
	No	1		1	
Maternal smoking	Yes	1.94 (1.00-3.79)	0.052	5.14 (1.78-14.80)	0.002
	No	1		1	

Factors affecting PEFR: linear regression

We found that the weight of the child and duration of breastfeeding were negatively associated with PEFR. On the other hand, age, any associated allergy, history of maternal asthma, paternal smoking, and parents' education were positively associated with PEFR for the overall sample.

Among cases, a positive association was found for the male sex, the child's height, and history of paternal asthma and maternal smoking with PEFR. However, the frequency of breastfeeding was found to be negatively associated. Similarly, among controls, male sex, the height of the child, history of sibling asthma, and maternal smoking had a positive association. At the same time, duration and frequency of breastfeeding were negatively associated, as shown in Table 5.

**Table 5:** Linear regression model to determine the predictors of PEFR among study participants. ^a^ Model R-squared (adjusted) = 0.958; p < 0.01. ^b^ Model R-squared (adjusted) for cases = 0.988; p < 0.01. ​​​​​​​^c^ Model R-squared (adjusted) for controls = 0.996; p < 0.01.

	Cases		Controls		Total	
Predictor ^a, b, c^	Incidence (B)	p-value	Incidence (B)	p-value	Incidence (B)	p-value
Male sex	10.78	<0.01	11.821	<0.01	4.16	0.294
Weight (kg)	0.45	0.557	−0.627	0.355	−2.46	0.046
Age (years)	4.55	0.429	−3.238	0.536	24.93	0.009
Duration of breastfeeding (months)	−0.53	0.123	−1.175	<0.01	−3.03	<0.01
Height (cm)	2.35	0.002	2.704	<0.01	0.18	0.878
Associated allergy	−5.16	0.210	−5.797	0.538	28.87	<0.01
Paternal asthma	−21.48	<0.01	−4.270	0.386	1.13	0.881
Sibling asthma	−2.10	0.695	−12.489	0.002	−4.89	0.506
Maternal asthma	1.92	0.678	1.250	0.895	34.32	<0.01
Paternal smoking	0.22	0.957	−2.138	0.393	13.84	0.005
Maternal smoking	10.07	0.016	13.145	0.001	−3.35	0.621
Parents’ education (literate)	1.53	0.744	−5.520	0.241	21.33	0.003
Term birth	−6.77	0.097	1.815	0.601	3.41	0.602
Breastfeeding frequency						
Never breastfed	−253.80	<0.01	−152.937	0.006	−74.72	0.467
<6 months	−239.84	<0.01	−122.180	0.027	−51.87	0.608
6 months or more	−229.39	<0.01	−118.524	0.033	−62.13	0.542

## Discussion

The beneficial effects of breastfeeding on a baby as well as the mother are non-arguable. However, the role of breastfeeding in reducing the risk of childhood asthma has remained controversial for a long time. Therefore, we conducted this matched case-control study to explore the association between breastfeeding and asthma along with PEFR.

We observed a protective association between breastfeeding and asthma. The median duration of breastfeeding among children with asthma was five (2.5-10) months as compared to children without asthma [9 (3.5-16.8) months]. In terms of odds ratio, the odds of the duration of breastfeeding among cases was 0.13 times or 13% lower than controls [0.87 (0.79-0.94)]. Also, the odds of non-exclusive breastfeeding were 0.85 or 85% [1.85 (1.08-3.16)] more among children with asthma than in the control group. Many cohort, case-control, and cross-sectional studies have reported protective associations of breastfeeding and asthma till the early 21st century [15-19]. A few studies did not confirm this association at a similar time frame [11-13]. After 2006, we could not find a single primary study even conducted to resolve this grey area, yet the most important research domain. Though three systematic reviews and meta-analyses have been carried out with similar intentions as ours in the last decade, they carry their limitations owing to primary studies included in the reviews [10,20,21].

A recent meta-analysis by Dogaru et al. in 2014 reported similar results of a positive association between breastfeeding and reduction in the development of asthma or wheezing. They also reported that the results are independent of study design and settings [20]. A similar review conducted by Lodge et al. (2015) did not find any significant association between exclusive breastfeeding for longer than three to four months and asthma at 5-18 years. Heterogeneity among the studies was very high [OR 0.94 (0.69-1.29), I2 = 81%]. However, they found a protective effect of breastfeeding for asthma among children who were ever breastfed compared to never breastfed, again leading to inconclusive evidence even from the reviews. In such cases, the evidence from primary studies is always warranted [21].

We found that many parental factors like their education level, paternal and maternal smoking, history of maternal asthma, and any other associated allergy were found to be significantly associated with the development of asthma among cases and controls apart from breastfeeding. It was found that the odds of parents being literate were lower among cases than controls [OR 0.09 (0.03-0.27)]. While there are a host of factors that may contribute to this, it is highly probable that the mothers' education, particularly regarding the disease and its manifestation, plays a role in the disposition of the mothers to ensure that the child has a prolonged period of breastfeeding [22].

Also, the odds of maternal asthma [21.4 (4.22-109.36)], paternal smoking [1.44 (0.22-0.86)], and maternal smoking [5.14 (1.78-14.80)] were higher among children with asthma as compared to children without asthma. This corroborates the results obtained from the studies conducted by Kull et al. (2002) and Karmaus et al. (2008) [18,23]. Their findings revealed that exclusive breastfeeding places children from mothers with no smoking history at 45% less risk [OR 1.491 (0.41-5.40)].

The median PEFR among cases was significantly less among cases [126 (113-141.8) l/min] than in controls [197.5 (176-214) l/min], with a p-value of <0.01, indicating that PEFR is a measure of differentiation between asthmatic cases and normal controls. During our analysis, we considered PEFR as one of the predictors and did the conditional logistic regression. However, the adjusted odds ratio which was computed was far from reality. After removing PEFR from the analysis, the regression could be done. That indicated the possibility of PEFR to be an effect modifier rather than a predictor for asthma.

We found that the weight of the child and duration of breastfeeding were negatively associated with PEFR. On the other hand, age, any associated allergy, positive maternal asthma, paternal smoking, and parents' education was positively associated with PEFR for the overall sample. Factors like age, height, and weight have been sufficiently explored to correlate with PEFR positively [24-26]. At the same time, we found a negative association between PEFR and the child's weight, which contradicts most studies. Another relationship of PEFR with breastfeeding has not been studied much, especially among children with asthma. Banerjee et al. reported that prolonged breastfeeding is associated with increased PEFR among healthy pre-pubertal boys [25]. However, we found a negative association between the duration of breastfeeding and PEFR in healthy children and the overall sample. Moreover, no association of duration of breastfeeding was found among any cases. This is one crucial prospective research area for clinicians.

This study has several important implications, both from a policy and research perspective. It was found that the prevalence of exclusive breastfeeding for at least six months among mothers residing in the cantonment was only 60% for the complete sample of 360 children (50% among cases and 69% among controls), which is similar to a national average of 55% as per a recent report of Poshan (2017) [27]. It was also found that the prevalence of maternal smoking was about 12%, which is an under-researched area in India due to cultural factors. Both these factors are significantly associated with the development of asthma, breastfeeding being protective and maternal smoking as deteriorative. Both these factors call for immediate attention through behavior change communication models and health promotion strategies, possibly due to the army's lifestyle norms. The focus should be to promote breastfeeding and discourage maternal and paternal smoking from reducing the risk of asthma development.

The government of India has extended a mandatory six months paid maternity leave to work mothers. Despite that, the prevalence of exclusive breastfeeding is way below the WHO standards. This highlights the need for research to explore the potential factors which hamper exclusive breastfeeding for six months. We found a negative correlation between duration and breastfeeding frequency with PEFR, which is also one grey area of medical research. This domain needs to be understood for explaining this bizarre finding.

As it was a case-control study, the recall bias was inherent in the study design, which is a limitation, especially for assessing exposure. We could not explore some exposures like the administration of antibiotics to the mother during the prenatal period. Also, we could not distinguish between children that may have been breastfed exclusively for more than six months. Such analysis could have revealed the effects of breastfeeding at intervals such as nine and 12 months.

## Conclusions

To conclude, the prevalence of exclusive breastfeeding was 60% among children residing in the cantonment area. Prolonged and exclusive breastfeeding was found to be a protective factor against the development of asthma. Promotion of breastfeeding and smoking cessation should be a priority in the control of childhood asthma. Hence exclusive breastfeeding for at least six months, as recommended by WHO, is essential and supported by evidence. Further research should be conducted to explore the negative correlation between duration and frequency of breastfeeding and PEFR.

## Appendices

The standard questionnaire used to collect the data is provided in Table 6.

**Table 6:** Standard questionnaire used to measure exposure. The given format is used for the collection of information for the study, from the selected participants as mentioned in the materials and methods section. PEFR, peak expiratory flow rate.

Case No.:	Date:
Sl. No.	Particulars/questions
1	Name of the child: ___________________
2	Sex: ___________________
3	Age: ___________________
4	Weight (in kg): ___________________
5	Date of birth: ___________________
6	Height (in cm): ___________________
7	Monthly family income (in rupees) : ___________________
8	Parents education: ___________________
9	Socioeconomic status: ___________________
10	Was he/she breastfed or not: ___________________
11	If yes, duration of breastfeeding: ___________________
12	Family history: ___________________
13	Mother: asthmatic/healthy: ___________________
14	Father: asthmatic/healthy: ___________________
15	Sibling: asthmatic/healthy: ___________________
16	PEFR value (in L/min) (highest of three recordings): ___________________
